# Video-assisted thoracoscopic surgery for Andersson lesion in ankylosing spondylitis: A case report and literature review

**DOI:** 10.1097/MD.0000000000035378

**Published:** 2023-09-22

**Authors:** Wei-Xin Dong, Zhen-Tao Chu, Yong Hu

**Affiliations:** a Department of Spinal Surgery, Ningbo No.6 Hospital, Ningbo, Zhejiang Province, China.

**Keywords:** Andersson lesion, ankylosing spondylitis, surgery, thoracoscopic

## Abstract

**Rationale:**

Andersson lesion (AL), a phenomenon initially described by Andersson nearly 80 years ago, has been the subject of extensive research and various treatment modalities. The ongoing debate surrounding the need for anterior surgery in AL cases has spurred numerous proposed approaches. Despite the demonstrated efficacy of anterior surgery in achieving fusion and stabilization, its implementation is associated with prolonged operation time and heightened intraoperative bleeding.

**Patient concerns:**

A 32-year-old male patient presented at our hospital in February 2019 with a 2-month history of bilateral lower extremity weakness and sensory disturbances. These symptoms were exacerbated by a recent fall.

**Diagnosis:**

AL conbined with ankylosing spondylitis.

**Interventions:**

A 1-stage posterior fixation and decompression procedure was performed to ensure spinal stability, minimize deformities, and reduce surgical trauma. To achieve these goals, a 2-stage approach was employed, which included video-assisted thoracoscope-guided vertebrectomy, spinal canal decompression, and bone graft fusion.

**Outcomes:**

No recurrences of significant pain, limb numbness, or other symptoms were reported, ultimately leading to an improved quality of life for the patient.

**Lessons:**

We utilized video-assisted thoracoscopic surgery technology for anterior bone graft fusion in a patient with AL to minimize the trauma of secondary surgery. However, the 3-year follow-up showed insufficient bony fusion at the fracture site. Nevertheless, the patient maintained spinal stability with posterior internal fixation and no significant kyphosis or symptoms. Thus, standalone posterior fixation may suffice for favorable clinical outcomes in patients with AL.

## 1. Introduction

Andersson Lesion (AL), first reported by Andersson in 1937, refers to sclerosis and destruction around the disc margins in the lumbar and thoracic vertebrae, as observed in radiographs of patients. AL is a complication of advanced ankylosing spondylitis (AS) and is characterized by a destructive lesion at the intervertebral disc-vertebral body junction, primarily affecting the anterior, middle, and posterior columns.^[1]^ AL commonly occurs in the thoracolumbar region, leading to increased local pain, kyphosis, and potential nerve damage. The diagnostic criteria for AL are not well-defined, resulting in the use of various terms to describe these lesions in AS. Treatment approaches for AL vary, ranging from conservative measures to surgical interventions. Surgical options include anterior or posterior fixation fusion, as well as combined anterior-posterior fusion. This article presents a case study of AL in AS that was treated with posterior decompression followed by thoracoscopically assisted anterior fusion in a 2-stage procedure.

## 2. Case report

**Figure 1.**
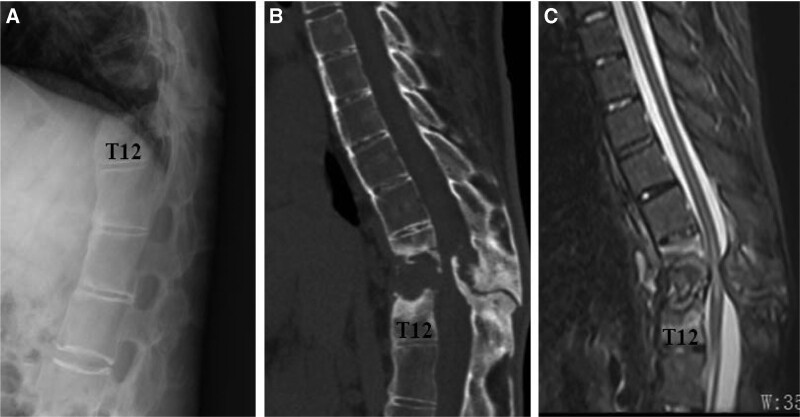
The X-ray (A), CT scan (B), and MRI (C) images depict syndesmophytes in the thoracic and lumbar regions, destruction of the T11 and T12 vertebrae, and extradural cord compression with myelopathic changes. CT = computed tomography, MRI = magnetic resonance imaging.

A 32-year-old male patient presented at our hospital in February 2019 with a 2-month history of bilateral lower extremity weakness and sensory disturbances. These symptoms were exacerbated by a recent fall. The patient also experienced difficulty walking and had compromised urinary and bowel functions. Previous conservative treatments, including rest, nonsteroidal anti-inflammatory drugs, and methylprednisolone, yielded no improvement. Physical examination revealed limited spinal mobility and tenderness over the T11 to 12 spine. Lower extremity muscle strength was approximately Grade II, accompanied by diminished sensation. Neurological examination showed a positive Babinski sign and ankle clonus, while the Lasegue sign was negative. The patient had a 10-year history of AS, with no prior tuberculosis, weight loss, fever, cough, or night sweats reported. Laboratory tests revealed a positive HLA-B27 result, an elevated erythrocyte sedimentation rate of 56 mm per hour, and a C-reactive protein level of 17.7 mg/L. Blood counts and other serum chemistries were within normal ranges. X-ray imaging indicated the presence of syndesmophytes in the thoracic and lumbar regions, as well as destruction of the T11 and T12 vertebrae. Computed tomography (CT) scan revealed increased bone density in the T11-T12 vertebral body and accessories, partial vertebral body absorption, and the presence of osteophytes within the spinal canal. Magnetic resonance imaging revealed abnormally elevated hyperintensity in the T11/12 disc, erosions of adjacent endplates, subchondral bone marrow edema, and extradural cord compression with myelopathic changes (Fig. 1). Based on these imaging findings and the patient’s medical history, a diagnosis of AS combined with a thoracic fracture and spinal cord injury was established.

Upon admission, the patient received comprehensive symptomatic treatment, including analgesia and neurotrophic support. Considering the patient’s medical history of AS, progressive symptom deterioration, and spinal cord impairment, the initial stage procedure was performed on February 16, 2019, under general anesthesia. This involved posterior pedicle screw fixation, debridement of the lesion, and subsequent anterior bone graft fusion using autologous iliac bone. The initial operation also included posterior thoracic decompression, fixation, fusion, and a biopsy of the lesion. Intraoperatively, a significant amount of scar tissue and chondroid hyperplasia were observed within the lesion. Histopathological analysis of the necrotic tissue revealed degenerative fibrocartilaginous tissue with fibrovascular proliferation and collagen accumulation. No evidence of epithelioid cell granulomas, Langhans giant cells, acid-fast bacilli, or malignant cells was found. Based on the patient’s medical history, clinical manifestations, imaging examinations, and pathological results, a definitive diagnosis of “AS with Andersson lesion” was confirmed.

**Figure 2.**
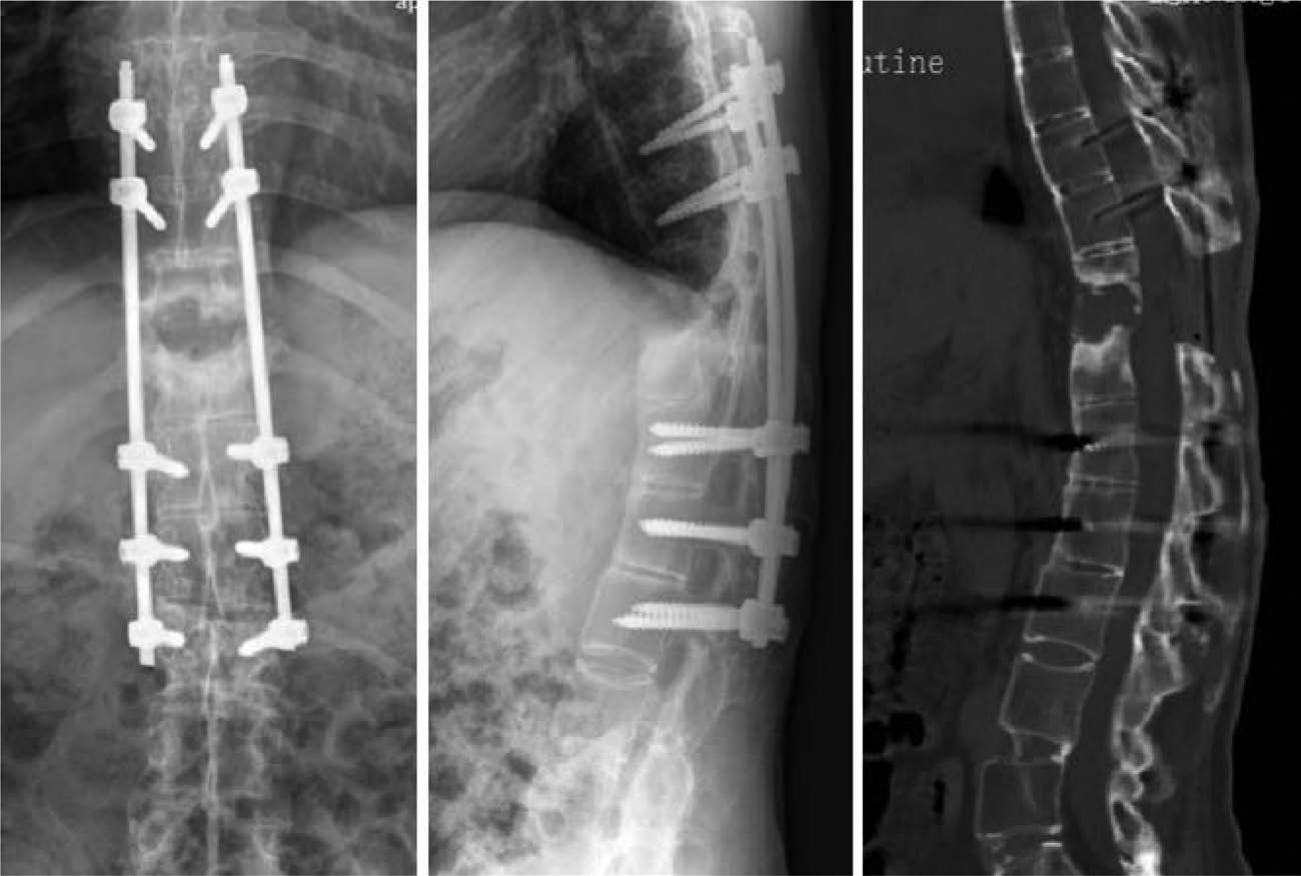
The radiograph and CT scan of the initial surgery demonstrate complete decompression, proper positioning of the internal fixation, and a significant anterior gap created by posterior fixation to correct the kyphosis. CT = computed tomography.

**Figure 3.**
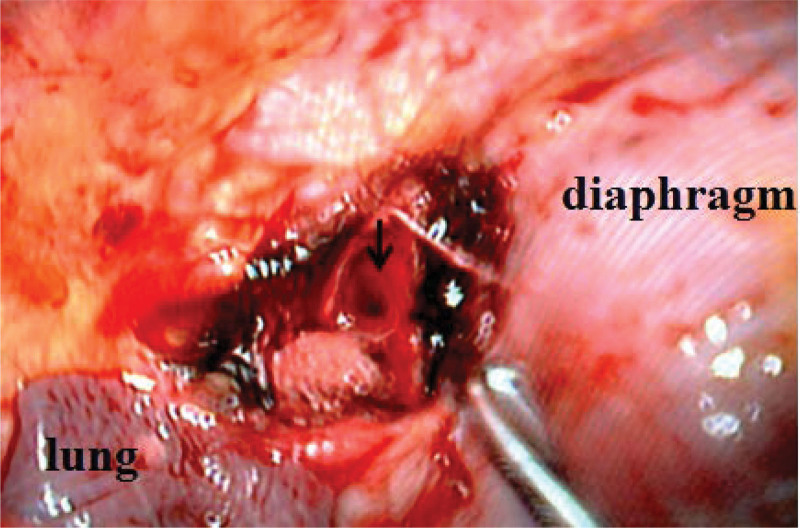
This image, captured during thoracoscopic surgery, highlights the placement of autologous ilium in the gap between the T11 and T12 vertebrae (indicated by the arrow).

**Figure 4.**
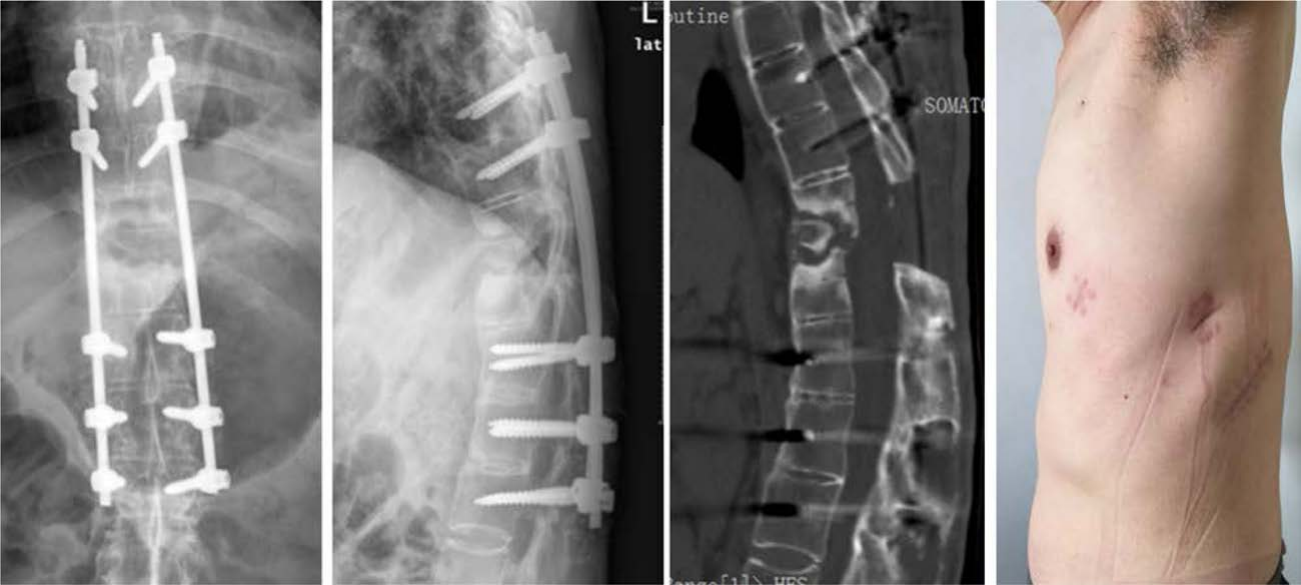
The radiograph, CT scan, and photograph illustrate the second-stage surgery involving anterior fusion, including the use of autologous ilium bone graft, with the assistance of a thoracoscope. CT = computed tomography.

**Figure 5.**
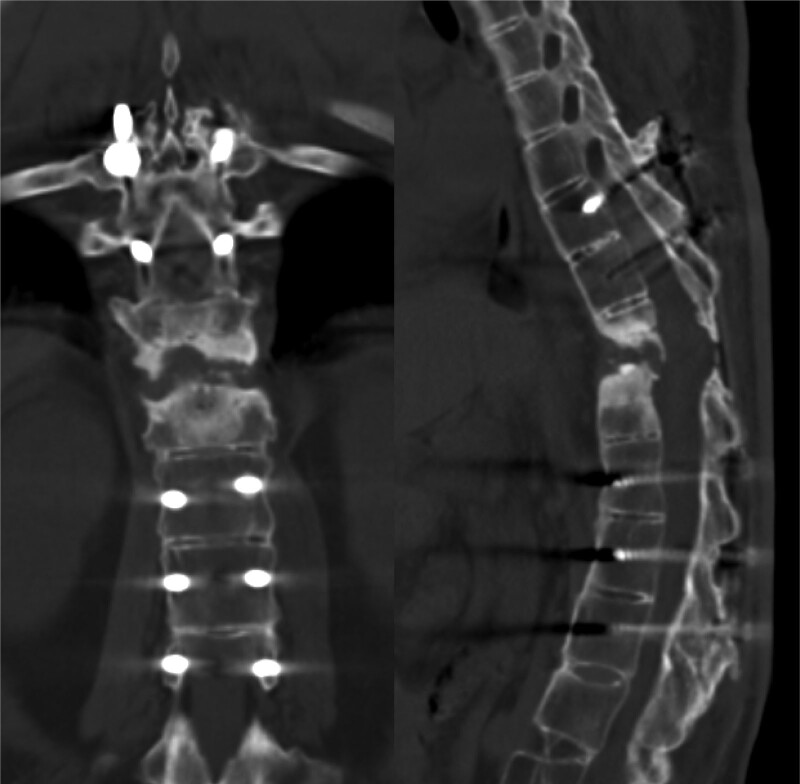
The imaging results from the three-year follow-up after surgery reveal excellent positioning of the internal fixation, along with osteophyte hyperplasia at the margin of the T11–T12 intervertebral gap and bone sclerosis at the fracture site.

Postoperatively, the patient experienced significant relief from low back pain, along with gradual improvement in lower limb numbness. Three days after the initial procedure, follow-up radiographs and CT scans confirmed complete decompression and proper positioning of the internal fixation. However, a considerable anterior gap was observed due to the posterior fixation used to correct the kyphosis (Fig. 2). Nine days after the first-stage procedure, the second-stage anterior fusion surgery was performed with the assistance of a thoracoscope. Autologous iliac bone graft was utilized to address the defect in the anterior and middle columns, promote bone fusion at the fracture site, and minimize surgical trauma (Figs. 3 and 4). The closed thoracic drainage tube was removed once the drainage flow significantly decreased, and subsequent chest X-rays or CT scans revealed no complications. The patient underwent regular outpatient follow-up examinations after discharge. Over the following 3 months, notable improvements in pain were observed, and the patient began walking with support. Muscle strength in both lower limbs recovered to Grade IV, and normal urination and defecation functions were restored. However, 3-year follow-up imaging post-surgery demonstrated excellent positioning of the internal fixation, accompanied by osteophyte hyperplasia at the margin of the T11 to T12 intervertebral gap and bone sclerosis at the fracture end (Fig. 5). No recurrences of significant pain, limb numbness, or other symptoms were reported, ultimately leading to an improved quality of life for the patient.

## 3. Dissussion

AL is a well-recognized complication of AS, with a reported prevalence ranging from 1.5% to over 28%.^[2]^ The pathogenesis of AL is believed to involve a combination of inflammatory processes and mechanical external forces that lead to tissue damage.^[3]^ In patients with AS, the inflammatory response and spinal fusion process are not evenly distributed along the spine during the course of the disease. The inflammatory reaction between fused vertebrae weakens the stability of the rigid, ossified spine, resulting in localized micromotion and impaired bone fusion.^[4]^ Furthermore, Bron et al^[5]^ have reported that advanced stages of AS are characterized by vertebral osteoporosis and spinal fusion, further compromising the structural integrity of the spine. Moreover, the thoracolumbar segment, particularly the junction between the costal joint and the lower lumbar spine, is subjected to significant mechanical stress. Even minimal or no external forces can lead to stress fractures. This finding may explain the occurrence of the T11 to T12 fracture in the patient following the fall.

When AS is accompanied by AL and the lesion remains localized, conservative treatment options such as bed rest and bracing can be employed to promote solid fusion of the lesion between the vertebrae. However, the presence of a “long tubular” ossified stiff spine in the upper and lower regions of the fractured segment, which experiences similar leverage, hinders the natural healing of AL and often leads to suboptimal outcomes with conservative management.^[6]^ Surgical intervention becomes necessary when conservative treatment fails or when symptoms worsen, such as in cases of intolerable pain, progressive symptomatology, worsening kyphotic deformity, or neurological deficits.^[5]^ The surgical approach aims to decompress the spinal canal and restore spinal stability. The ongoing debate on surgical treatment primarily revolves around the necessity of anterior bone graft and fusion. Liang et al^[7]^ conducted a retrospective study involving 14 AS patients with severe kyphosis and AL who underwent posterior wedge osteotomy via a posterior approach, resulting in kyphosis correction. Ling et al^[8]^ presented a case series of 11 patients with AS-related AL who underwent one-stage posterior osteotomy and bone graft fusion, achieving successful local fusion and favorable clinical outcomes. Similarly, Wang et al^[4]^ reported the effectiveness of surgical treatment involving both posterior fixation and anterior fusion. However, Qian et al^[9]^ argue that a single posterior approach for correction and internal fixation may lead to noticeable bone defects in the anterior and middle columns of the affected vertebra. These defects in the anterior and middle columns could increase local stress on the posterior internal fixation system and elevate the risk of vertebral instability, potentially resulting in complications such as loosening of the internal fixation or even breakage of screws and rods.

In our perspective, the selection of the surgical procedure depends on various factors, including the localization of the lesion, spinal alignment, presence of neurological deficits, and the site of compression. In this case, the patient presented with progressive neurological deficits and a kyphotic deformity, and the lesion was situated in the thoracolumbar spine, resulting in a 3-column injury. Following a one-stage posterior operation, a significant anterior gap was created by the posterior fixation to correct the kyphosis, and the edges of the gap exhibited sclerosis. Consequently, we opted for anterior fusion in this patient. For the anterior fusion procedure, we chose video-assisted thoracoscopic surgery (VATS), which was initially introduced by Mack^[10]^ for the treatment of thoracic disc herniation in 1993. As reported by Arunakul,^[11]^ advancements in technology and improved posterior surgical techniques have led to a decrease in the frequency of thoracoscopic anterior release, particularly after reaching its peak between 2000 and 2002. The anterior release procedure has largely been replaced by posterior-only techniques. However, VATS still offers recognized advantages over thoracotomy, including reduced trauma to the chest wall tissues, decreased postoperative pain, and lower complication rates.^[12]^ In our patient, whose costovertebral joint had fused, VATS provided excellent visualization of anatomical structures through small incisions. Nonetheless, in this case, the imaging examination conducted 3 years after the operation revealed incomplete bony fusion following the anterior bone grafting. However, the position of the posterior internal fixation was satisfactory, and there were no indications of intervertebral space collapse or progressive kyphosis. We analyzed the reasons for the inadequate bony fusion as follows: The limited space available during VATS anterior surgery posed challenges in dealing with complex vertebral lesions and completely removing necrotic tissue; There was a significant defect in the anterior and middle columns of the vertebrae, and the performed anterior bone grafting was insufficient. The implanted bone remained unconnected to the fractured end; The combined anterior and posterior approaches significantly impacted the blood supply to the vertebra.

## 4. Conclusion

In this case, we employed VATS technology for anterior bone graft fusion in a patient with AS and AL, aiming to minimize the trauma associated with secondary surgery. However, the results of the 3-year follow-up revealed inadequate bony fusion at the fracture site. Nevertheless, the patient maintained spinal stability through posterior internal fixation without significant kyphosis or symptoms. The authors suggest that the use of a titanium mesh cage may be superior to iliac crest autograft in cases with substantial defects in the anterior and middle columns.

## Author contributions

**Data curation:** Wei-Xin Dong.

**Formal analysis:** Zhen-Tao Chu.

**Investigation:** Wei-Xin Dong, Zhen-Tao Chu.

**Writing – original draft:** Wei-Xin Dong.

**Writing – review & editing:** Yong Hu.
